# Species conservation profile and revision of *Salviakorolkowii* (Lamiaceae, Lamiales), a narrow endemic of the Western Tian-Shan

**DOI:** 10.3897/BDJ.10.e89437

**Published:** 2022-08-12

**Authors:** Alexander Sennikov, Obidjon Turdiboev

**Affiliations:** 1 University of Helsinki, Helsinki, Finland University of Helsinki Helsinki Finland; 2 Institute of Botany, Tashkent, Uzbekistan Institute of Botany Tashkent Uzbekistan

**Keywords:** Central Asia, conservation, IUCN assessment, Kazakhstan, Kyrgyzstan, Labiatae, Mountains of Central Asia biodiversity hotspot, Tian-Shan, Uzbekistan

## Abstract

**Background:**

*Salviakorolkowii* (Lamiaceae) has been considered a species of highest conservation priority due to its narrow distribution and isolated taxonomic position. The species has been known from Uzbekistan and, questionably, Kyrgyzstan and treated as endemic to the Western Tian-Shan. Its modern conservation status according to the IUCN Criteria has not been established.

**New information:**

The taxonomic position of *Salviakorolkowii* is evaluated; the species is treated as the sole member of S.sect.Odontochilus (Pobed.) Sennikov, comb. nov. because of its morphological differences and phylogenetic isolation. The herbarium collections are completely revised and the species is proven to occur mainly in Uzbekistan with a single locality (new country record) in Kazakhstan; its former report from Kyrgyzstan (one locality) is confirmed and documented by herbarium specimens. The species occurrences are mapped and its conservation status is assessed as Vulnerable due to the restricted size and continuous decline of its populations because of the ongoing degradation and destruction of its primary habitat (variegated outcrops) at lower elevations. This study highlights the importance of a thorough revision of herbarium collections in Central Asia for conservation purposes.

## Introduction

The genus *Salvia* L. (Lamiaceae Martinov, Mentheae Dumort., Salviinae Endl.), encompassing nearly 1000 species (Kriebel et al. 2019), has undergone marked species radiations in three regions of the world: Central and South America, Central Asia/Mediterranean and Eastern Asia, where it colonised different biomes and adapted to different pollinators (Drew et al. 2017, Hu et al. 2018, Kriebel et al. 2019). A broad-scale phylogeny of the genus was developed and demonstrated that the genus comprises three major clades, which largely correspond to the Mediterranean and south-western Asian, eastern Asian and New World centres of its species diversity (Kriebel et al. 2019). Over a half of its total species diversity falls into the New World, which is a secondary radiation area, whereas the ancestral area for the genus radiation was placed to the Irano-Turanian floristic region, which is the second great centre of its species diversity (Walker et al. 2004, Kriebel et al. 2019).

Recent phylogenetic studies (Drew et al. 2017, Hu et al. 2018) argued that a broad circumscription of *Salvia* is preferable, although an opposite view has been advocated by some researchers (Will and Claßen-Bockhoff 2017). In a broad sense, *Salvia* is characterised by two anterior fertile stamens, with two thecae on each stamen (one theca may be sterile) separated by a variably elongated connective tissue, whereas the other genera of Mentheae possess four fertile stamens (Drew et al. 2017 and references therein). In this circumscription, the broad *Salvia* includes some small genera traditionally recognised in Central Asia and the former USSR (e.g. *Perovskia* Kar., *Rosmarinus* L.), which are deeply embedded within *Salvia* on the phylogenetic tree (Drew et al. 2017) and, therefore, abandoned in our latest synoptic revisions (Tojibaev et al. 2021). Morphological differences between major phylogenetic clades of *Salvia* are largely limited to the characters of the elongated connective tissue that separates thecae in stamens, are at least partly driven by changes between different pollinators (Drew et al. 2017) and evolved parallel variants of the staminal architecture independently in each part of the world (e.g. Fragoso-Martínez et al. 2018).

*Salvia* is well represented in the mountainous regions of Central Asia. Its species grow on mountain slopes and outcrops of variegated beds, in foothills and the lower mountain belt. According to Makhmedov (1984a), 21 and 11 species of *Salvia* in its traditional circumscription occur in the Pamir-Alai Mountains and in the Western Tian-Shan, respectively. The expanded *Salvia* counted 41 species in Central Asia (Turdiboev et al. 2022), although this figure did not take into account the synonymy proposed by Lazkov (2016) and Tojibaev et al. (2021) and should, therefore, be corrected. *Salviakorolkowii* Regel & Schmalh. (Regel 1879), *S.tianschanica* Makhm. (Makhmedov 1980) and *S.vvedenskii* Nikitina (Nikitina 1962) of the Western Tian-Shan are currently listed in the Red Data Books of Kyrgyzstan (Mosolova 2006) and Uzbekistan (Tojibaev 2009) as rare and subendemic plants.

Being part of the Mountains of Central Asia biodiversity hotspot (Körner 2004, Spehn et al. 2005), the Western Tian-Shan is rich in plant diversity (3440 species and subspecies) and endemic plants (634 taxa) (Tojibaev et al. 2021). *Salviakorolkowii* is endemic to the Western Tian-Shan (Tojibaev et al. 2020) and occurs in rare and scattered populations. The species has potential ornamental and medicinal values (Mamadalieva et al. 2017).

While working on the Flora of Uzbekistan project (Sennikov et al. 2016), we revised herbarium specimens of *Salvia* from Central Asia stored at TASH and LE. During this revision, we have found that the published data on *S.korolkowii* are highly incomplete and its conservation assessment is badly outdated. Although this species has been legally protected for nearly 50 years, its distribution remains poorly researched and its taxonomic position has not been updated since Pobedimova (1954). Its latest regional revision (Lazkov 2016) highlighted a number of open questions, which should be resolved for a modern conservation assessment under the IUCN.

The aim of the present contribution is to resolve historical and nomenclatural problems connected with *S.korolkowii* and to re-assess the distribution and conservation status of this species, which has been under the highest conservation priority in Kyrgyzstan (Sharashova 1985, Mosolova 2006) and Uzbekistan (Makhmedov 1984b, Tojibaev 2009) since the times of the USSR (Takhtajan 1975, Takhtajan 1981, Makhmedov and Denisova 1984), due to its restricted distribution area and isolated taxonomic position.

## Material and methods

The data about *Salviakorolkowii* (history of discovery, distribution, ecology, morphology, taxonomic position) were collected from literature, herbarium collections and online depositories of published observations.

The nomenclature was re-assessed on the basis of the protologue (Regel 1879) and historical literature (Lipsky 1905), according to the International Code of Nomenclature of algae, fungi and plants (Turland et al. 2018). Original collections were examined at the Komarov Botanical Institute of Russian Academy of Sciences (LE).

Morphological characters were examined from taxonomic revisions (Pobedimova 1954, Lazkov 2016). The phylogenetic position and taxonomy was assessed on the basis of phylogenetic (Drew et al. 2017, Will and Claßen-Bockhoff 2017, Hu et al. 2018) and taxonomic (Kudryashev 1937, Pobedimova 1954, Makhmedov 1984a) literature.

A distributional dataset (Turdiboev and Sennikov 2022) is based on our comprehensive *de visu* revision of herbarium specimens kept at LE and TASH; several smaller collections of Central Asian plants (at E, H, MW, NL, NSK, P, PE, TALL and ZT) were examined online. The specimen data were complemented with documented observations (Plantarium 2022). The occurrences were georeferenced with the help of printed maps adjusted with Google Maps, using the utmost accuracy available; historical records from the same locality were linked to a single occurrence when no further precision was available (Usmonov et al. 2021). Place names were recorded according to the national languages (Kazakh, Kyrgyz, Uzbek); their spelling adopted here may deviate from the international tradition which is based on the Russian spelling.

To assess the threat status of the species, IUCN Red List Categories and Criteria were employed (IUCN 2012). The geospatial analysis was performed using the Geospatial Conservation Assessment Web Tool GeoCAT (Bachman et al. 2011). The AOO was based on a recommended cell width of 2 km.

## Results

### Historical background

The first difficulty connected with the name *Salviakorolkowii* is bibliographic. This species was described by Eduard Regel in a series of his numerous contributions of new species described on the basis of dried and living plant collections of the Imperial Botanical Garden in Saint-Petersburg. His contribution (fasciculus) 7 appeared in part 2 of volume 6 of the main periodical of the garden, *Acta Horti Petropolitani* (Regel 1880). At that time, due to the massive size of contributions, the journal appeared in bulky volumes consisting of a few large-sized parts, usually with three or more papers in each. In such cases, production and printing was rather slow and the volume parts were inconvenient in private circulation; for this reason, many significant papers were printed separately and distributed privately by the authors, whereas the journal volumes were intended for library use. For practical reasons, individual papers were typically preprinted immediately after typesetting by the same commercial printer (brothers Schumacher for volume 6), rather than reprinted from journal volumes, thus pre-dating the journal publication and distribution. The preprints were separately paginated, omitting the journal pagination, whereas the typeset remained identical. For this reason, bibliographically, the instalments of Regel’s *Descriptionesplantarumnovarum* may be treated as books rather than articles in periodicals and the nomenclatural novelties published in that series may be cited from those books rather from the journals. The time difference between preprints and journal volumes (or their parts) was sufficiently short to fall within the same calendar year, making the distinction rather impractical. However, the situation was different for Regel’s fasciculus 7 (Regel 1879), which was printed and distributed in 1879, whereas its volume part was dated 1880. This difference is significant and should be taken into account.

*Salviakorolkowii* Regel & Schmalhausen (Regel 1879: 70) was described with an indication of its provenance in Latin: “In Turkestania occidentali prope Chodschent 3000’ alt.; inter Ak-tag-tau et Ak Cagoti, 5–8000’ alt. (Korolkow)”. The protologue states that the plants were collected by a Russian military officer in Turkestan, Colonel (thereafter General) Nikolai I. Korolkov near Xuçand (also spelled Khodzhent in Russian) Town, now in northern Tajikistan, probably implying the southern part of the Kurama Range. Kudryashev (1937) suggested that this indication is a spelling mistake and the locality should rather read Xo'jakent (also spelled Khodzhakent), now in eastern Uzbekistan, situated along Chirchiq River between the Qorjontov Range and the Chatqol Range. This conclusion is confirmed by the analysis of Korolkov’s herbarium collections published by Lipsky (1905), who found that Korolkov collected near Burchmullo Village at the stated elevation, and by the other collections cited in Regel (1879), where "Chodschent" is clearly linked with Burchmullo. Climatic conditions in the two locations differ (Kudryashev 1937) and, according to the published data, *S.korolkowii* does not occur at Xuçand (Zhogoleva and Kochkareva 1986).

The other locality mentioned in the protologue is situated near Chimgon (Chimyon), very close to Xo'jakent and Burchmullo. As noted by Lipsky (1905), Korolkov collected near Chimgon and Xo'jakent in June of 1872, travelling between Chimgon (Chimyon) Mt. and Oqsoqotasoy River. The toponyms mentioned in the protologue can be interpreted as follows: Ak-tag-tau = Oqtaxta Pass, Ak Cagoti = Oqsoqotasoy River; this apparently denotes the upper course of the river, which starts under that pass.

This means that all the original localities of *S.korolkowii* are situated in the mountains surrounded by the Chirchiq, Chatqol, Oqsoqotasoy and Teraklisoy Rivers (Chatqol Range, Western Tian-Shan), within the area of ca. 30 km in diameter. Among the collections examined at LE, only one gathering (collected between Oqsoqotasoy and Oqtaxta) was found. The species nomenclature is as follows.

***Salviakorolkowii*** Regel & Schmalh., Trudy Imp. S.-Peterburgsk. Bot. Sada 6: 356 [prepr. 70] (1879) ≡ *Schraderiakorolkowii* (Regel & Schmalh.) Pobed. in Schischkin, Fl. URSS 21: 373 (1954) ≡ *Arischradakorolkowii* (Regel & Schmalh.) Pobed., Novit. Syst. Pl. Vasc. 9: 247 (1972) ≡ *Stiefiakorolkowii* (Regel & Schmalh.) Soják, Čas. Nár. Mus., Odd. Přír. 152(1): 22 (1983).

Type: UZBEKISTAN. "Inter Ak-tag-tau et Ak Cagoti, 5–8000’ alt." [between Oqsoqotasoy River and Oqtaxta Pass, 1500-2400 m a.s.l.], June 1872, *Korolkoff s.n.* (lectotype LE0051698, designated by Lazkov (2016); isolectotypes LE0051697, LE01072812, K000929822 and P02864044).

The species name was spelled "Korolkowi" in the protologue. This spelling is correctable to "korolkowii" according to Art. 60.8(b). In modern databases, the correct spelling was adopted in the Leipzig Catalogue of Vascular Plants (Freiberg et al. 2020, Freiberg 2020), whereas the World Checklist of Vascular Plants (Govaerts et al. 2021, The Royal Botanic Gardens, Kew 2021) used the wrong spelling "korolkovii".

When designating the lectotype, Lazkov (2016) did not specify the particular sheet because of the lack of appropriate identifiers at that time. The lectotype specimen was clearly labelled as "Type" and, therefore, Lazkov's lectotype designation was unambiguous and effective.

### Taxonomic classification

The taxonomic position of *Salviakorolkowii* varied with time, when researchers realised that the early attempts to circumscribe and subdivide the genus *Salvia* were unnatural and, therefore, unsatisfactory. This species was originally assigned to S.sect.Hymenosphace Benth. (Regel 1879), which was treated very broadly in those times. The most extreme splitting of *Salvia* was upheld by Nevski (1937), who accepted many minor generic segregates including the genus *Schraderia* Medik. Pobedimova (1954) followed this approach and classified *Salviakorolkowii* in *Schraderia*. When *Schradera* Vahl was conserved against its near-homonym *Schraderia* Medik., Pobedimova (1972) renamed this segregate to *Arischrada* Pobed. At the next twist, Soják (1983) returned to the old nomenclature of Medikus (1791) and found another suitable generic name, thus renaming the species as *Stiefiakorolkowii* (Regel & Schmalh.) Soják.

The phylogeny of *Salvia* (Dizkirici et al. 2015, Will and Claßen-Bockhoff 2017) revealed a broad paraphyly of the traditional S.sect.Hymenosphace, in which *S.korolkowii* was retained by Makhmedov (1984a). Bentham (1833b) originally included 12 species into this section, of which *S.pomifera* L., *S.canariensis* L., *S.aurea* L. and *S.paniculata* L. were listed in its protologue (Bentham 1833a).

Pobedimova (Pobedimova 1954, Pobedimova 1972) understood that the broad S.sect.Hymenosphace was too diverse and artificial. She subdivided this taxon (as *Schraderia*) into smaller, more natural groups. The species of this affinity occurring in Central Asia, *S.bucharica* Popov and *S.korolkowii*, she placed in separate sections, which were subsequently downgraded to subsections (Makhmedov 1982, Makhmedov 1984a, Lazkov 2016, Turdiboev and Turginov 2021). The relationship and separate positions of these sections were recently confirmed (Will and Claßen-Bockhoff 2017).

The sections differ in the following morphological characters (Pobedimova 1954): *Holochilus* (*S.bucharica*) – upper lip nearly entire, flowers pink or purple, floral leaves persistent, leaves pinnatisect, upper thecae connected and fertile; *Odontochilus* (*S.korolkowii*): upper lip tridentate, flowers yellow, floral leaves caducous, leaves simple, upper thecae free and sterile.

The correct type species of S.sect.Hymenosphace is *S.pomifera*, which was designated by Makhmedov (1982). It is clear that the Central Asian species previously placed into S.sect.Hymenosphace are not closely related to any species originally assigned to this section (Will and Claßen-Bockhoff 2017). For this reason, the system of narrowly defined sections proposed by Pobedimova (Pobedimova 1954, Pobedimova 1972) can be retained and transferred to the nomenclature of *Salvia* as elaborated here.

***Salvia*** sect. ***Holochilus*** (Pobed.) Sennikov, **comb. nov.** ≡ Schraderiasect.Holochilus Pobed. in Schischkin, Fl. URSS 21: 664 (1954) ≡ Arischradasect.Holochilus (Pobed.) Pobed., Novit. Syst. Pl. Vasc. 9: 247 (1972). Type: *Salviabucharica* Popov. Species: *Salviabucharica* Popov, *S.hydrangea* Benth., *S.maymanica* Hedge.

***Salvia*** sect. ***Odontochilus*** (Pobed.) Sennikov, **comb. nov.** ≡ Schraderiasect.Odontochilus Pobed. in Schischkin, Fl. URSS 21: 664 (1954) ≡ Arischradasect.Odontochilus (Pobed.) Pobed., Novit. Syst. Pl. Vasc. 9: 247 (1972) ≡ Salviasubsect.Odontochilus (Pobed.) Makhm., Bot. Mater. Gerb. Inst. Bot. Akad. Nauk Uzbeksk. S.S.R. 20: 30 (1982). Type: *Salviakorolkowii* Regel & Schmalh. Monotypic section.

### Distribution area

The species was considered endangered due to its narrow distribution area (Makhmedov and Denisova 1984, Makhmedov 1984b). For this reason, it was mapped already in the 1980s (Makhmedov 1984a), but that mapping was extremely coarse and incomplete.

The species distribution is apparently centred in Uzbekistan, where the greatest majority of its localities are situated (Turdiboev and Sennikov 2022). Its occurrence in Kyrgyzstan (in "Chatkal River basin") was postulated (Sharashova 1960) seemingly because of the close proximity of localities in Uzbekistan to the border with Kyrgyzstan, but no specimens have been known to date (Mosolova 2006, Lazkov 2016). We found two specimens of *Salviakorolkowii* at LE which were collected in Kyrgyzstan by R. Kamelin in 1972, who unknowingly entered the territory from the neighbouring Uzbekistan along the Chatqol River and collected the plants within 0.5-2 km from the border. These are the first documented records of the species from Kyrgyzstan.

During our inventory of herbarium collections, quite unexpectedly we found one overlooked specimen at TASH, which was collected in 1924 near Tūrbat Village in Türkıstan Region of Kazakhstan. This specimen documents the first record of *S.korolkowii* from the country.

According to our data, the localities of *S.korolkowii* are concentrated along tributaries of the Chirchiq River: Oqsoqotasoy, G'alvasoy, Qorangko'lsoy, Chatqol, Oqbuloq, Koʻksuv, Piskom and Ugom, with isolated localities along the Boshqizilsoy River and at Tūrbat Village. This area is situated within the Chatqol, Ugom, Koʻksuv, Piskom and Qorjontov mountain ranges.

*Salviakorolkowii* occurs at lower elevations, mostly between 900 and 1200 m, but sometimes up to 1500-1700 m above sea level. In the Western Tian-Shan, lower elevations are very hot and highly arid; in this territory, the species prefers open stony substrates of red clay and sandstone mixed with gypsaceous sediments, which are called variegated denudations due to their alternation of red sandstone and white gypsum patches. This landscape is very rich in narrowly distributed plants (Popov 1923, Kamelin 2017) and seems to be a major contributor to the highest plant endemism recorded in the Western Tian-Shan (Tojibaev et al. 2021).

Makhmedov (1984b) noted that many populations of *S.korolkowii* are small but some may be significant in size. Due to its availability, the species was distributed to international scientific herbarium collections as exsiccata, collected along the Koʻksuv (Makhmedov 1988) and Piskom (Pobedimova 1967) Rivers in 50 duplicates.

## Species Conservation Profiles

### Salvia korolkowii

#### Species information

Scientific name: Salviakorolkowii

Species authority: Regel & Schmalh.

Synonyms: *Schraderiakorolkowii* (Regel & Schmalh.) Pobed., *Arischradakorolkowii* (Regel & Schmalh.) Pobed., *Stiefiakorolkowii* (Regel & Schmalh.) Soják.

Common names: Корольков мармараги (Uzbek Cyrillic), Шалфей Королькова (Russian), Корольков көк башы (Kyrgyz), Корольков сәлбен (Kazakh Cyrillic).

Kingdom: Plantae

Phylum: Spermatophyta

Class: Magnoliopsida

Order: Lamiales

Family: Lamiaceae

Taxonomic notes: The species belongs to a presumably monotypic section, Salviasect.Odontochilus (Pobed.) Sennikov. Its closest relative in Central Asia is *S.bucharica* Popov. (S.sect.Holochilus (Pobed.) Sennikov).

**Figure 1.**
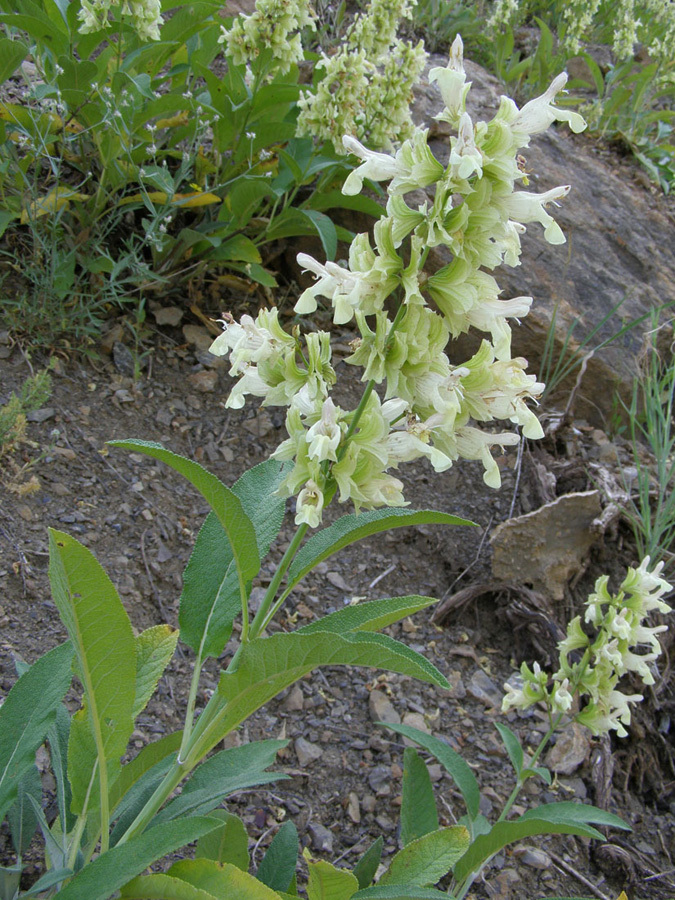
A plant of *Salviakorolkowii* at Xumson, Uzbekistan. Photographed by A. Gaziev, 1 June 2008. Source: https://www.plantarium.ru/page/image/id/48037.html.

**Figure 2.**
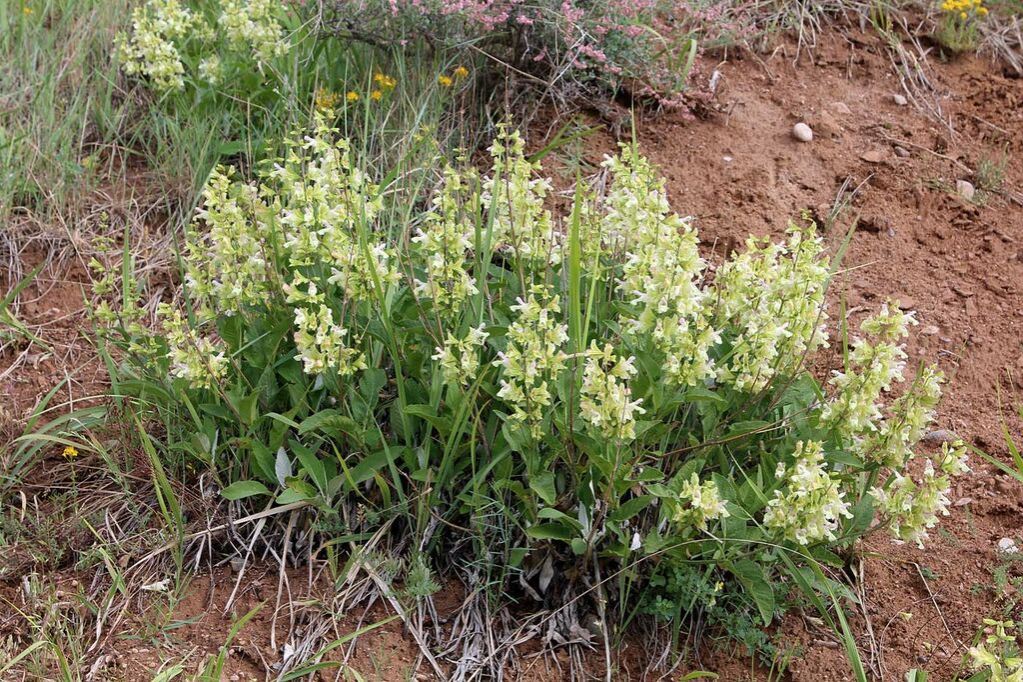
A large cushion of *Salviakorolkowii* along the Beldorsoy River, Uzbekistan. Photographed by A. Gaziev, 8 June 2014. Source: https://www.plantarium.ru/page/image/id/246310.html.

Figure(s) or Photo(s): Figs 1, 2

Region for assessment: Global

#### Editor & Reviewers

##### Reviewers

Reviewers: Lazkov, G.A.

##### Editor

Editor: Sennikov, A.N. & Turdiboev, O.A.

#### Geographic range

Biogeographic realm: Palearctic

Countries: UzbekistanKazakhstanKyrgyzstan

**Figure 3.**
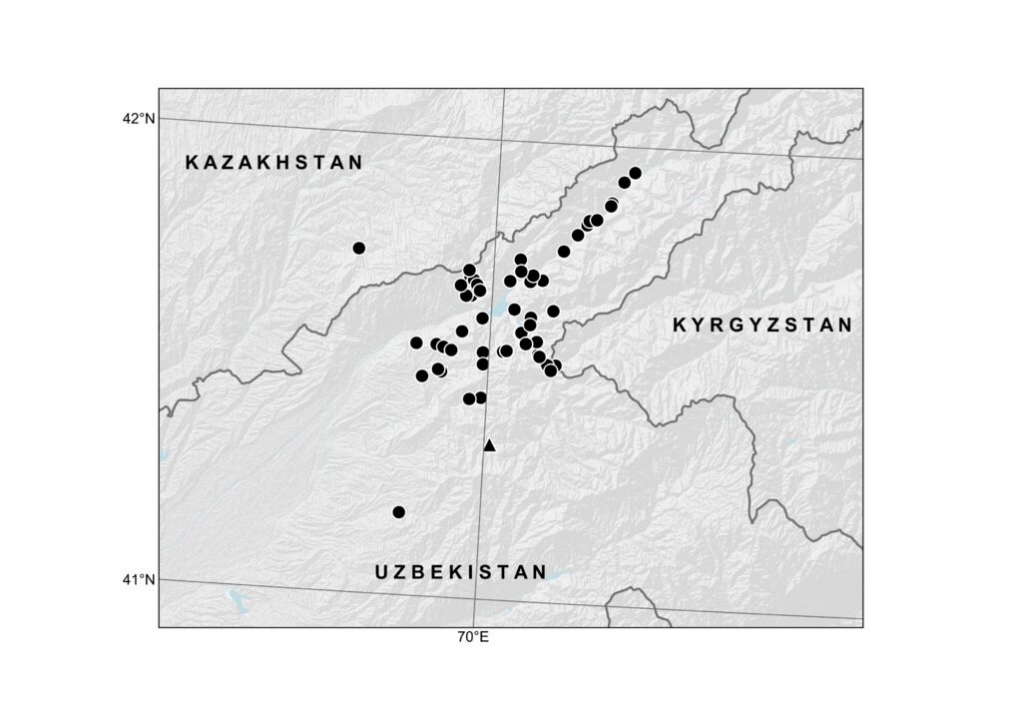
Distribution area of *Salviakorolkowii* according to the specimens examined and documented observations. Symbols: triangle - lectotype locality, dots - other records.

Map of records (image): Fig. 3

Map of records (Google Earth): Suppl. material 1

Basis of EOO and AOO: Observed

Basis (narrative): The species is known from several populations. It was estimated that ca. 200 local populations may exist in Uzbekistan (Makhmedov 1984b), where the species forms a compact core distribution area ca. 65 km in diam. with many documented localities, from which it has been repeatedly sampled for 150 years (Turdiboev and Sennikov 2022). The species is highly conspicuous, easy to detect and identify and tends to occur in easy-to-access places; for this reason, we assume that the major part of its distribution has been revealed and most of its populations are known. Our data inventory has expanded the total known distribution area (Tojibaev 2009) by ca. 30 km only.

Min Elevation/Depth (m): 850

Max Elevation/Depth (m): 1700

Range description: The species habitat is mountainous, occurring along the Chirchiq River and its tributaries (Oqsoqotasoy, G'alvasoy, Qorangko'lsoy, Chatqol, Oqbuloq, Koʻksuv, Piskom and Ugom), in the Chatqol, Ugom, Koʻksuv, Piskom and Qorjontov mountain ranges. The distribution area lies mostly in Uzbekistan, with a minor penetration into Kyrgyzstan and one isolated locality in Kazakhstan.The species occurs in highly arid territories, mostly between 900 and 1200 m, but sometimes up to 1500-1700 m above sea level.

#### Extent of occurrence

EOO (km2): 2800

Trend: Stable

Justification for trend: Although the species is under high anthropogenic pressure because of many of its populations being situated in the immediate vicinity of populated places or roads, which may lead to destruction or impoverishing of some populations, there is no evidence that any part of its distribution area is lost.

Causes ceased?: No

Causes understood?: Yes

Causes reversible?: No

Extreme fluctuations?: No

#### Area of occupancy

Trend: Decline (observed)

Justification for trend: Most of the localities of *Salviakorolkowii* in Uzbekistan are situated in the nearest proximity to populated places, roads and other areas of human activity. The species occurs mostly in the lower mountain belt, in the lower part of river valleys. Such locations are most easily accessible and susceptible to residential and recreational development, road construction and land use, especially close to the Chirchiq River; as an example, several populations were destroyed when the territory was inundated by the Chorvoq water reservoir (Makhmedov 1984b).The most significant populations of the species are known along the Chatqol, Piskom and Ugom Rivers (Makhmedov 1984b, Turdiboev and Sennikov 2022). The species is still regularly encountered in accessible places along the Chirchiq and Chatqol Rivers (Plantarium 2022, Turdiboev and Sennikov 2022) despite the anthropogenic pressure.

Causes ceased?: No

Causes understood?: Yes

Causes reversible?: No

Extreme fluctuations?: No

AOO (km2): 192

#### Locations

Number of locations: 25

Justification for number of locations: The species is documented from a number of localities, which can be treated as about 25 locations, based on the most significant threatening event, i.e. habitat destruction by large-scale actions (inundation, road construction, mining, development).

Trend: Decline (observed)

Justification for trend: At least one location was nearly destroyed under the Chorvoq water reservoir.

Extreme fluctuations?: No

#### Population

Number of individuals: Estimated as less than 10000 mature individuals.

Trend: Decline (observed)

Justification for trend: The species is under constant pressure due to large-scale activities that lead to irreversible habitat destruction and degradation, and some of these activities (inundation, road construction, development) are known to reduce the number of individuals, although the extent of this reduction cannot be estimated with certainty.

Basis for decline: (a) direct observation

Causes ceased?: No

Causes understood?: Yes

Causes reversible?: No

Extreme fluctuations?: No

Population Information (Narrative): The exact population size and the number of individuals are unknown. The count provided by Makhmedov (1984b) is based on extrapolations.

#### Subpopulations

Abundance largest subpopulation: 1000

Number of subpopulations: 25

Trend: Decline (inferred)

Justification for trend: Some subpopulations situated at lower elevations close to populated places are under ongoing threat because of urban development and road construction and may disappear or noticeably degrade in the future.

Extreme fluctuations?: No

Severe fragmentation?: No

#### Habitat

System: Terrestrial

Habitat specialist: Yes

Habitat (narrative): The species often grows on variegated beds, which are situated at elevations of ca. 900-1200 m a.s.l. This habitat is highly arid, much insolated and very warm; its vegetation cover is naturally very sparse.More seldom, the species occurs also at higher elevations of 1500-1700 m a.s.l., in sparse steppoid plant communities on rocky slopes.

Trend in extent, area or quality?: Decline (observed)

Justification for trend: Variegated beds are highly prone to destruction because of their situation at lower elevations and close proximity to populated places. Habitat loss occurs due to expansion of populated places, road construction, human changes in watercourses etc.

##### Habitat

Habitat importance: Major Importance

Habitats: 6. Rocky areas (e.g. inland cliffs, mountain peaks)

#### Ecology

Size: 30-50 cm

Generation length (yr): 30

Dependency of single sp?: No

Ecology and traits (narrative): The plants are subshrubs forming a long vertical root and a strong branching caudex which is capable to last for several decades. Shoots bicyclic, developing a rosette in the first year and a leafy stem in the second year. Leaves long-petiolate, blades narrowly oblong in rosettes and ovate-oblong on stems, white-tomentose on the lower side. Flowering stems are very showy because of conspicuous yellow flowers 2.5-3 cm long, which are collected in large thyrsoid inflorescences. Reproduction exclusively by seed.

#### Threats

Justification for threats: The species populations are situated at lower elevations and in the lower part of river ravines. For this reason, many populations are situated next to populated places and along roads and have been in danger because of continuous residential development and road construction. Some complementary threat comes from grazing, sports and recreation, which may damage individual plants.In the past (1960s), some populations were destroyed by the construction of the Chorvoq water reservoir.

##### Threats

Threat type: Ongoing

Threats: 1.1. Residential & commercial development - Housing & urban areas1.3. Residential & commercial development - Tourism & recreation areas2.3.2. Agriculture & aquaculture - Livestock farming & ranching - Small-holder grazing, ranching or farming4.1. Transportation & service corridors - Roads & railroads

##### Threats

Threat type: Past

Threats: 7.2. Natural system modifications - Dams & water management/use

#### Conservation

Justification for conservation actions: The species is not protected in any strict nature reserve, but its distribution area is largely covered by the Ugom-Chatqol National Park (Uzbekistan), in which some lands are protected, whereas economic activities and recreation are regulated. The only locality in Kazakhstan is situated within the Sairam-Ögem National Park.Cultivated in the Tashkent Botanical Garden. Seeds have not been submitted to the Millennium Seed Bank or any similar institution.The species is included in the Red Data Books of Uzbekistan (Tojibaev 2009) and Kyrgyzstan (Mosolova 2006) as a rare and vulnerable plant and is, therefore, under legal protection even outside strictly protected areas.Using the IUCN Criteria (rather small population size and continuous decline), we suggest the global conservation status of *Salviakorolkovii* to be assessed as Vulnerable (criteria C1+2a(i)).

##### Conservation actions

Conservation action type: In Place

Conservation actions: 1.1. Land/water protection - Site/area protection5.1.2. Law & policy - Legislation - National level

#### Other

Justification for use and trade: Despite its limited distribution, the species was considered as a source of essential and drying oils tested as a component of varnish (Zuckerwanik and Grachev 1948). The actual use is prevented by legal protection.Although the plants are not picked up by local people and tourists, the species has a potential value as ornamental for plant enthusiasts. Such actual use is unknown.There has been no noticeable harvest of the species, except for scientific purposes. The species is no longer collected for scientific distribution either.The species is cultivated ex-situ in the Tashkent Botanical Garden.

##### Use and trade

Use type: National

Use and trade: 5. Manufacturing chemicals13. Pets/display animals, horticulture16. Establishing ex-situ production *

##### Ecosystem services

Ecosystem service type: Less important

##### Research needed

Research needed: 1.2. Research - Population size, distribution & trends3.1. Monitoring - Population trends

Justification for research needed: Although we believe that the major part of the distribution area of *Salviakorolkowii* has been already detected, its exact localities and population size have not been properly studied and documented.

## IUCN Red List assessment

Due to its restricted extent of occurrence (2800 km^2^) and area of occupancy (190 km^2^), the species may qualify for Endangered, but we estimate the number of its locations as ca. 25. Its total population size is estimated as less than 10000 individuals and the projected loss is estimated at 10% within the future 100 years; for this reason, we assess its global threatened category as Vulnerable (criteria C1+2a(i)).

IUCN Red List assessment

## Conclusions

The endemic flora of the Western Tian-Shan is very rich (Tojibaev et al. 2021) but has never been assessed for conservation purposes in its entirety. Modern inventories have been recently started with selected species of *Cousinia* (Usmonov et al. 2021) and *Tulipa* (Wilson et al. 2021). This effort should be continued to cover other species of the area with restricted distributions.

*Salviakorolkowii* has been legally protected since the 1970s in the USSR, based on the draft assessment of R.V.Kamelin who considered its isolated taxonomic position, showy habit and very narrow distribution area (Takhtajan 1975). This protection continues in present-day Kyrgyzstan (Mosolova 2006) and Uzbekistan (Tojibaev 2009). Although the species does not occur in any strictly protected area, its distribution is largely covered by national parks in Kazakhstan and Uzbekistan, thus ensuring some protection for its native landscapes from large-scale destruction. However, many of its populations are under continuous threat of reduction or even extinction due to their proximity to populated places and communications, which makes the species vulnerable as assessed here.

Our study highlights the importance of herbarium collections in the botanical research of Central Asia. Even in cases of such well-known and unambiguous species as *Salviakorolkowii*, which have been in conservation focus for decades, historical data may contain some important but cryptic information which have never been evaluated and included in scientific works. Given the small size of distribution areas of many plants of the Western Tian-Shan, precise and comprehensive data inventory and mobilisation is required to overcome the obscurity and complexity of historical collections, which are often dispersed among various countries and require a special effort in their deciphering and evaluation (e.g. Sennikov 2021).

Conclusions

## Supplementary Material

B8DA814C-C100-53E1-88DF-15A0EF827BC9 10.3897/BDJ.10.e89437.suppl1 Supplementary material 1 Distributional data for *Salviakorolkowii* Data type occurrences Brief description Distribution data based on the comprehensive revision of herbarium specimens and published human observations, as used in GeoCAT. Detailed information: https://doi.org/10.15468/7j3uer. File: oo_692172.kml https://binary.pensoft.net/file/692172 Turdiboev, O.A. & Sennikov, A.N.
